# Varieties of Mobility Measures: Comparing Survey and Mobile Phone Data during the COVID-19 Pandemic

**DOI:** 10.1093/poq/nfac042

**Published:** 2022-12-02

**Authors:** Fabian Kalleitner, David W Schiestl, Georg Heiler

**Affiliations:** PhD Candidate, Department of Economic Sociology, University of Vienna, Vienna, Austria; PhD Candidate, Department of Economic Sociology, University of Vienna, Vienna, Austria; PhD Candidate, Complexity Science Hub Vienna, Vienna, Austria, and Institute of Information Systems Engineering, Technical University Wien, Vienna, Austria

## Abstract

Human mobility has become a major variable of interest during the COVID-19 pandemic and central to policy decisions all around the world. To measure individual mobility, research relies on a variety of indicators that commonly stem from two main data sources: survey self-reports and behavioral mobility data from mobile phones. However, little is known about how mobility from survey self-reports relates to popular mobility estimates using data from the Global System for Mobile Communications (GSM) and the Global Positioning System (GPS). Spanning March 2020 until April 2021, this study compares self-reported mobility from a panel survey in Austria to aggregated mobility estimates utilizing (1) GSM data and (2) Google’s GPS-based Community Mobility Reports. Our analyses show that correlations in mobility changes over time are high, both in general and when comparing subgroups by age, gender, and mobility category. However, while these trends are similar, the size of relative mobility changes over time differs substantially between different mobility estimates. Overall, while our findings suggest that these mobility estimates manage to capture similar latent variables, especially when focusing on changes in mobility over time, researchers should be aware of the specific form of mobility different data sources capture.

## Introduction

Since the start of the COVID-19 pandemic, social scientific research has contributed immensely to the understanding of human behavior in times of a global health crisis (Bavel et al. 2020). One crucial aspect in this endeavour is the analysis of human mobility (Chu et al. 2020; Haug et al. 2020). Empirical studies analyzing the consequences of non-pharmaceutical interventions such as school closings or retail closings have been used extensively to inform governments about the effectiveness of these policies (Alfano and Ercolano 2020; Schlosser et al. 2020; Vinceti et al. 2020). At the same time, researchers began to investigate the micro-level mechanisms explaining mobility changes to enable governments to tailor measures more effectively (e.g., Pfattheicher et al. 2020; Heffner, Vives, and FeldmanHall 2021).

These research goals produced two rather separate strands in the literature in which scholars typically follow one of two distinct approaches to measure mobility. First, social scientists interested in micro-level mechanisms mostly rely on surveys asking individuals to self-report their mobility. This individual-level data allows researchers to study important covariates or implement experimental manipulations aiming to uncover the social mechanisms that explain the (un)effectiveness of certain policy changes (Jordan, Yoeli, and Rand 2021; Kittel, Kalleitner, and Schiestl 2021). However, using self-reports to estimate individuals’ mobility also entails several drawbacks: next to the high expenses of surveys, self-reports include the risk of survey specific measurement errors (e.g., social desirability and recall bias) and are limited in their measurement frequency (Schwarz and Oyserman 2001).

Second, new technological innovations enabled researchers to trace people’s actual behaviors using mobility data from mobile phones on a large scale. These data were obtained either directly from service providers or indirectly via third-party sources and make use of data from users’ device sensors (such as the Global Positioning System [GPS]) or from the mobile phone network (e.g., based on Global System for Mobile [GSM] communications) (Buckee et al. 2020; Grantz et al. 2020; Oliver et al. 2020). However, while these data are cost-effective and carry the potential to cover large areas (Zhang et al. 2022), they often lack information on potential explanatory variables of mobility and face potential coverage issues.

Despite differential advantages, so far, only a limited number of studies combine “sensor data” and survey data (Gollwitzer et al. 2022; Kitamura and Yamada 2020). This might partially be explained by issues like data protection, data availability, and limited experience with these new methods in social survey research (Struminskaya et al. 2020). For these reasons, two rather distinct fields of COVID-19 mobility research emerged, either using mobile phone data or using survey data. This opens a gap in the literature, as we do not know whether commonly used mobility measures using survey self-reports behave similarly to those using behavioral data from mobile phones.

Several potential sources of representation and measurement errors could produce differences in mobility estimates depending on the data source used: users’ limited ability to self-report their mobility behavior in surveys renders detailed questions on the traveled distances impossible and may introduce social desirability bias and recall bias (Schwarz 2007). Contrary to that, data based on device sensors (such as GPS) offer high positioning accuracy and measurement frequency but may face coverage errors because only a part of the population owns a smartphone and nonparticipation error because of the limited number of users willing to participate in passive mobile data collection (Rojas, Sadeghvaziri, and Jin 2016; Keusch et al. 2019). Compared to data from device sensors, data based on GSM communications commonly comprise a larger number of users but are less exact in positioning (Gauvin et al. 2020).

The present article compares mobility trajectories across these three data sources using datasets that capture mobility patterns in Austria over a long period: Google’s (GPS-based) Community Mobility Reports, aggregated GSM mobility data from a large Austrian mobile service provider, and self-reported mobility from a representative web-based panel survey. Specifically, the paper investigates similarities and differences of the three data types in aggregate relative changes in mobility trends over time.

## Data and Methods

For our survey estimates, we use data from a non-probability web-survey panel provided by the Austrian Corona Panel Project (ACPP) (Kittel et al. 2020a, 2020b). The panel consists of approximately 1,500 participants per wave in 22 waves, spanning March 2020 until April 2021. Survey respondents were recruited from a preexisting commercial online access panel. Respondents needed to reside in Austria, be at least 14 years old, and have access to the internet. Respondents were invited based on a quota sample by age, region, municipality size, and education. Survey waves were fielded on a weekly (waves 1–10), bi-weekly (waves 11–14), and monthly basis (waves 15–22). To measure mobility, we calculate an additive index summarizing the answers to questions about the frequency respondents left their homes in the preceding 7 days for the following reasons: work, sports, meeting friends or relatives, buying medicine or receiving medical treatments, buying food, buying non-food products, walking pets, boredom, and other reasons (respondents could answer on a 5-point rating scale ranging from never to daily). Because few respondents are older than 74 (3.01 percent), we limit our sample to those younger than 75. Applying listwise deletion of missing values, we receive 27,491 observations from 2,685 unique individuals. We apply poststratification weights so that the sample represents the Austrian population between 14 and 75 years. Question wordings and further details on the survey are given in Appendix A and B. Additional details on the construction of the additive index and analyses to test nonresponse and panel attrition are presented in Supplementary Material section A.

Second, we use data on Austrian-wide mobility during the COVID-19 pandemic observed from mobile phones based on the location of GSM network base stations they are assigned to. Anonymized data from the GSM network was provided by a large Austrian mobile service provider (MSP). Our analyses are based on aggregated data containing daily mobility estimates by age group, gender, and region. These aggregates rely on a micro-dataset that registers events for every call as well as data communications of approximately 1.2 million devices registered with the partner MSP (Heiler et al. 2020). Local data privacy regulations have been met, and the recommendations of the alliance of mobile phone providers have been followed. To estimate individual mobility, we use the radius of gyration (ROG), which is defined as the time-weighted root mean square distance between the center of gravity (i.e., the coordinate-wise time-weighted average) and each localization. This measure is commonly used to estimate mobility patterns of the general population (González et al. 2008; Song et al. 2010; Pappalardo et al. 2015). The distribution of the ROG is heavily skewed. To mitigate the effect of data skew, we use the daily median ROG to calculate weekly aggregated mobility averages. As the GSM data includes information about the age group and gender of its users, we also compare GSM and survey self-reports within and between these subgroups.

We weight the data so that the resulting distributions resemble the Austrian target population (aged 15–74). This approach enables us to exclude the possibility that differences in the distribution of these variables between the GSM mobility data and the survey data bias our mobility estimates. For further details on the GSM data and the calculation of ROG refer to Appendix A.

Third, we use data from the COVID-19 Community Mobility Reports published by Google (Google 2021a). This dataset provides aggregated daily movement trends by region across six different types of places: grocery stores and pharmacies, parks, transit stations, retail and recreation areas, residential areas, and workplaces. Google specifies that these reports are created with aggregated, anonymized sets of data from users who have turned on the “Location History” setting. Besides GPS data, Google’s Location History service uses further information derived from WiFi and mobile network. However, Google provides no further details on the measure itself except that it describes people’s “visits and length of stay” (Google 2021b). Thus, this study also provides a benchmark for how this measure compares to other mobility estimates. Google mobility data is reported as a percent change to a baseline, which is the median value of the five weeks from January 3 to February 6, 2020. We calculate an additive index of all six different mobility categories and estimate weekly averages. The differentiation of places also allows us to compare specific mobility trends to specific self-reported frequencies in the survey. To do that, we calculate an additional additive index summing survey answers on the frequency of “buying medicine or receiving medical treatments” and “buying food” and compare it to the mobility reported in Google’s “grocery & pharmacy” category. Furthermore, we compare survey answers on “buying non-food products” to Google’s “retail & recreation” category and “work” to Google’s “workplace” category.

We focus on the detection of mobility trends, representing one of the main goals in mobility research during the pandemic, and compare them between data sources. Due to the heterogeneous measurements and scales, a direct comparison of these mobility estimates is challenging. Hence, this paper investigates similarities and differences of the three data types in aggregate relative changes in mobility trends over time. We define the first wave of the survey as our baseline week (March 23–29, 2020) and calculate relative percentage changes from this baseline for all three data sources. Afterward, we match weekly mobility averages in the GSM and Google data to the respective survey waves (we provide additional checks calculating a 7-day moving average and varying the baseline in Supplementary Material  sections A and B). Thus, we compare relative changes in the countrywide aggregates of self-reported mobility, GSM mobility, and Google’s mobility reports. To test the similarity of aggregated mobility trends between different mobility estimates, we calculate Pearson’s correlations and trend lines. In addition, we provide robustness checks utilizing linear regression with time-fixed effects and a difference in difference analysis. We calculate all our analyses in R (R Core Team 2021). We use the package anesrake (Pasek 2018) to calculate the weights of the GSM data (command *anesrake*) and srvyr (Ellis and Schneider 2021) for weighting the data and calculating the specific aggregates (*as_survey_design*). We use the base R command *lm* for the OLS regressions and *cor.test* to calculate correlation coefficients. Graphs are done using the package ggplot2 (Wickham 2019). Refer to the data availability section for a link to the code.

## Results

### General Trends across Different Mobility Estimates

**Figure 1.**
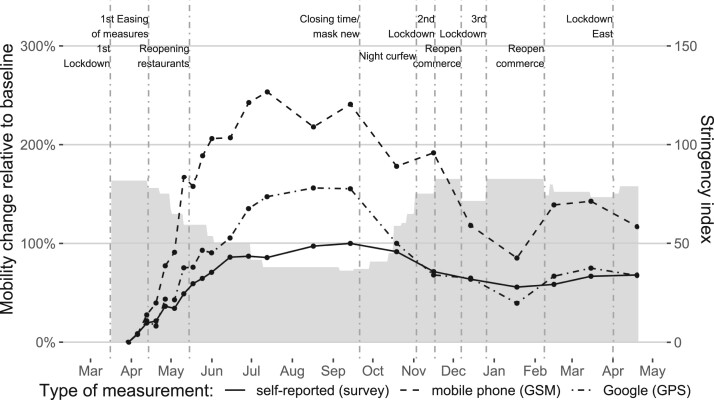
**Mobility trends in Austria.** The black lines represent average mobility changes relative to the baseline (Mar. 23–29, 2020). The gray area indicates the COVID-19 stringency index [0–100] (containment and closure policies) for Austria (Hale et al. 2021).

Figure 1 shows the trends in mobility patterns using three different sources to estimate mobility in Austria: GPS, GSM, and surveys. In general, trends of all three mobility estimates are quite similar. After the first lockdown, mobility rose until it reached its peak in summer 2020 (our baseline week is the last week of March 2020, one week after the beginning of the first lockdown in Austria). After the first restrictions had been reintroduced in September, mobility started to decrease. This decreasing trend had been relatively consistent until mid-January, when mobility slowly began to rise again. However, some differences between the estimates remain. The GSM estimated mobility pattern clearly shows the largest variation over time, which might be a result of the different measurement strategies: while the GSM-based mobility data estimates absolute physical mobility, the survey data comprise self-reported frequencies of certain types of mobility, resulting in the least amount of variation over time among the three measures. Google’s mobility estimates seem to capture a middle ground with more substantial variation than the survey estimates, but lower volatility than the absolute mobility estimates of the GSM data. This suggests that—in line with Google’s specifications—this estimate combines people’s frequencies of movements and duration of stay rather than measuring their (more volatile) absolute mobility.

### Mobility Trends within and between Subgroups

**Figure 2.**
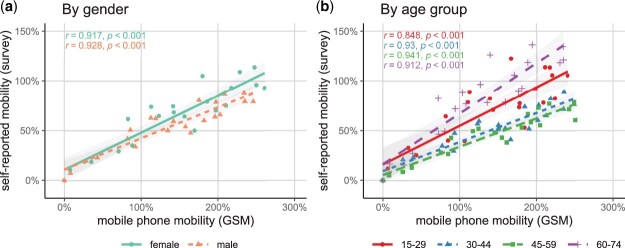
**Correlation of mobility estimates** (averages by subgroup and week). *P* values refer to two-sided tests for statistical significance.

To get a more nuanced picture of the similarities between the changes in survey mobility estimates and the two mobile phone-based mobility estimates over time, we focus on the correlations between these estimates. Pearson’s correlation coefficient provides a basic measure on whether aggregate mobility estimates within groups follow a similar time trend in both compared mobility measures. The high correlation between survey self-reports and estimated behavior from GSM data confirms the notion of strongly similar time trends (r = 0.927, *p* < 0.001, 2-sided). Figure 2 shows correlation coefficients remain high when differentiating by gender and age. The similar slopes in Panel (2a) indicate that differences in the mobility patterns between genders across data sources are consistent. This is not the case when looking at different age groups. The larger slopes of the fitted trend lines (2 b) for the youngest and the oldest respondents in the sample indicate that these groups show more variation in the survey estimates over time compared to middle-aged respondents (30 to 59 years). This is also supported by regression estimates reported in Supplementary Material  table S2. One explanation for these differences might be the stronger work obligations for middle-aged individuals: as work-related mobility was still permitted during the lockdown, workers could reduce their mobility less than other groups in society. This results in lower relative increases in the summer for this age group, especially within the survey data (see also relative changes over time in Supplementary Material section C). Differences between the estimates might emerge because increases in commuting should have smaller effects on the reported frequency of mobility in the survey estimate compared to the GSM estimate that focuses on absolute distances.

**Figure 3.**
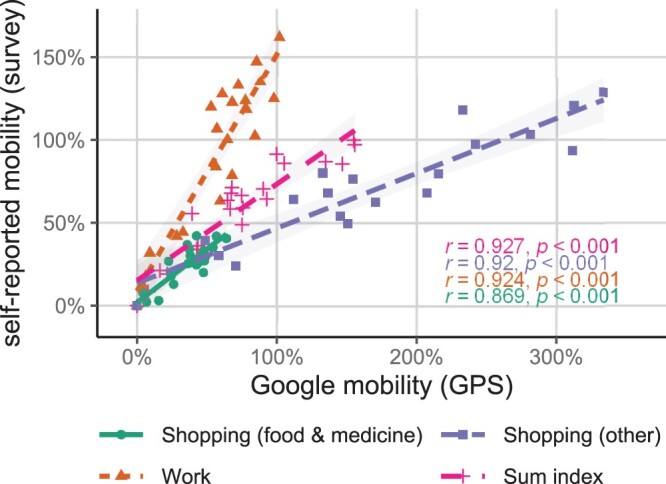
**Correlation of mobility estimates** (averages by category and week). *P* values refer to two-sided tests for statistical significance.

Figure 3 provides correlations between aggregate measures of frequencies on leaving the house for different reasons (self-reported) and the frequency of movement in different place categories (Google GPS). The correlation coefficients suggest high similarities within subgroup trends over time. However, the different slopes indicate different strengths in the relationships between the survey and Google’s mobility estimates. While the mobility estimate of the category “shopping (food and medicine)” nearly follows a 1:1 ratio, the mobility estimate of “shopping (other)” more than quadruples over time in the GPS estimates and only doubles in the survey estimate. Separate analyses for survey-related mobility to buy food or medicine suggest little differences in comparison to the combined index. On the contrary, work-related mobility increases by about 150 percent in the survey data, while the GPS estimate only doubles over time. Again, these results indicate substantial similarity in trends within subgroups, while differences between subgroups across the different mobility measures can be substantial. An important reason for this finding is the low amount of mobility in Google’s estimates related to “shopping (other)” (Google’s “retail & recreation” place category) during the start of the pandemic (see changes over time in Supplementary Material section C). Since takeaway and pick up were permitted during the lockdown, and Google’s mobility indicator also considers how long people stay in these places, the technology might underestimate shopping-related mobility in this period. Moreover, it is likely that Google’s place category captures a wider range of mobility than our corresponding survey estimate.

### Robustness Checks

To check the robustness of these results, we also provide a regression-based approach to compare the aggregated mobility estimates. These models use time-fixed effects and the estimates generally support the findings reported above (Supplementary Material  table S2). Furthermore, we calculated mobility patterns using a second mobility estimate that utilizes respondents’ self-reported frequency of staying at home. We compare these to GSM-data estimates indicating the share of people having an ROG of more than 500 meters. Overall, the results are qualitatively similar to those reported above (Supplementary Material section E). To further substantiate our finding that mobility patterns of these different estimates are qualitatively similar and capture at least closely related latent variables, we test the impact of a partial lockdown in Austria using a differences-in-differences approach. Results (reported in Supplementary Material section F) indicate that all three measures are capturing substantial decreases in mobility due to the lockdown.

## Discussion and Conclusion

Using data from Austria, this paper provides a comparison of mobility estimates from three main data sources: GPS, GSM, and survey data. Our findings show that all three mobility estimates change similarly over time and portray similar trends within socio-demographic subgroups. Although all three data sources might produce different and potentially biased point estimates of mobility due to differences in representation and measurement, researchers interested in over-time changes of mobility estimates should receive comparable results. Thus, surveys can indeed help to explain mobility patterns obtained from mobile phone data, and, vice versa, mobile phone data can substantiate self-reported behavior obtained from surveys.

Our findings support results in the literature suggesting that survey estimates measuring mobility and compliance with mobility restrictions show rather low levels of social desirability bias (Jensen 2020; Larsen et al. 2020; Daoust et al. 2021). In fact, when policy stringency decreases, we find that our self-reported mobility measure increases to a lesser degree than the other mobility estimates (see also Supplementary Material  table S2). Furthermore, using smartphone data directly linked to survey data, Gollwitzer and colleagues (2022) report high correlations between self-reported social distancing and actual movement data at both the individual and the state level. Our findings suggest that this result also holds over a long period spanning several different phases of the COVID-19 pandemic, and also persists when focusing on time trends specific to gender and age groups or different mobility categories.

However, our findings also highlight some differences: First, while trends are similar, the size of variation over time is not. Especially the GSM measure using data on absolute mobility produces more variation over time compared to the other mobility measures. Second, different mobility measures have different subgroup-specific variation over time. Our findings suggest that variation in work mobility over time should be more pronounced in the GSM measure, followed by the survey-based indicator, and finally by Google’s Mobility Reports. Hence, a simple comparison would suggest three different results for the relative importance of work for overall mobility changes during the COVID-19 pandemic. Researchers should be aware of the specific forms of mobility that different sources of mobility estimates capture and acknowledge differences between measures that target frequency of mobility, absolute mobility, or physical distance.

These issues of construct validity, targeting the “inferential leap” between the phenomenon of interest and the data source (Conrad, Keusch, and Schober 2021), are crucial besides the commonly mentioned issues of measurement and representation errors, which remain important even in Big Data contexts (Amaya, Biemer, and Kinyon 2020). However, despite specific sources of errors in GPS, GSM, and survey data sources, all seem capable of measuring mobility patterns during the COVID-19 pandemic. This highlights especially the power of research designs that rely on estimate changes over time. Nevertheless, more efforts in combining these measures at the micro level could enhance our understanding of how different data sources capture people’s behavior.

## Supplementary Material

nfac042_Supplementary_Data

## Data Availability

Replication data and documentation are partly available. The survey data are available at doi: 10.11587/28KQNS. Google mobility reports are available at: https://www.gstatic.com/covid19/mobility/Region_Mobility_Report_CSVs.zip. GSM data is not available because of the permission policy of the original data collector. The authors have asked the editors to waive the journal’s replication policy for this manuscript. Please contact the corresponding author for more information. The statistical code is available at https://doi.org/10.17605/OSF.IO/C6VN7.

## Appendix A. Further Details on the Data Sources and the Computation of Mobility Measures

### Survey Data

The panel survey provided by the Austrian Corona Panel Project (ACPP) uses non-probability sampling relying on a commercial online access panel provided by Marketagent.com (certified under ISO 20252). The target population includes Austrian residents at least 14 years of age and with access to the internet. The surveys were administered using web panel invitations sent out via email. One email reminder is sent 4 days after the initial invitation in each wave. The panel utilizes quota sampling based on the following variables and categories: gender (male, female); age (14–19, 20–29, 30–39, 40–49, 50–59, 60–69, 70–99); region (the nine Austrian provinces “Bundesländer”); municipality size (<5,000 inhabitants, 5,000–50,000, 50,000–1 million; >1 million); and highest level of education (ISCED 0–3; ISCED >3 = upper secondary education or higher). The original questionnaire was fielded in German. The survey has no information about respondent’s location while completing the survey. Thus, we cannot exclude respondents who are traveling away from home (answers to questions about the plans for summer holidays in 2020, however, suggest that less than 1.7 percent of respondents should be on vacation during each of the survey’s field periods).

The announced survey duration is 18 minutes and respondents receive 180 “credit points” as compensation after completing the survey, which can be exchanged to 1,80€ in cash or coupons. The initial “participation rate”, defined as the number of respondents who have provided a usable response divided by the total number of initial invitations (refer to AAPOR (2016, p. 49) for details), in the first survey wave was 35.2 percent (1,541 interviews after 4,381 invitations). On average, 1,547 respondents per wave completed the survey and panelists participated on average in 11.9 of the 22 survey waves. Participation rates to replace dropouts varied between 2.4 percent (July 2020) and 8.1 percent (May 2020). All who participated in the survey were invited to participate again in subsequent waves. Participation rates of subsequent waves started at 86.2 percent and dropped afterward, stabilizing after wave 12 at around 60 percent. On average, 58 participants per wave were invited to replace dropouts. Information on the field periods can be accessed on the original project website (ACPP 2021).

We use post-stratification weights that are provided alongside the official survey release. Weights were calculated using data about the distributions of gender, age groups, level of education, region, and gender by age group provided by the Austrian federal agency for statistics (Statistik Austria 2019). To verify that the same respondents answered each survey wave, basic socio-demographics (age, gender, region) were asked in every survey wave and cross-validated with the previous answers.

As a non-probability survey, the data has several limitations. First, statistical tests rely on the assumption that participants are randomly chosen from a defined set of participants (see, e.g., Vehovar 2016). Thus, the quality of the survey sample is dependent on the quality of panelists registered with the specific commercial online access panel in representing the target population. Marketagent.com has quite a large sample pool in Austria (129,500 registered panelists) and uses online and offline recruiting, including advertising on television, print media, radio, and billboards. This should reduce potential coverage error. Second, utilizing web mode only excludes part of the population by design. While still relevant to some degree, this issue has become less crucial in recent years, and coverage error should be smaller in our target group (14–74) compared to the general population (in 2021, 95 percent of Austrian households with at least one household member aged between 16 and 74 years had internet access) (Statistik Austria 2021). In sum, these issues suggest that the digitally skilled population might be overrepresented in our survey. This could make our survey-based mobility estimates more similar to the estimates provided by Google’s mobility reports and the GSM data compared to traditional probability-based surveys. Our main comparison focuses on change over time, thus consistency within the sample distribution should be most crucial. This highlights the issue of panel attrition which is discussed in Supplementary Material section A.

### GSM Data

The aggregated GSM data we use are based on micro data from a mobile service provider (MSP) located in the medium-to-high price segment for telephone and mobile internet services in Austrian business-to-consumer and business-to-business markets. The micro-dataset features a combination of classical CDR (Call Data Records) and XDR (X Data Records). Thus, the dataset not only registers an event when a call is performed but also includes additional events when data packages are transferred. On average the micro-dataset contains an event every 4 minutes for every user (this includes night times). For 80 percent of the events, the subsequent event is received within 1.7 minutes.

The 1.2 million devices are a sample of all customers of this MSP. The original dataset contains approximately one billion events per day from 4.5 million devices. Due to legal reasons, only those registered with the partner MSP as mobile handsets could be used. Thus, sensor devices from the Internet of Things as well as roamers and customers of virtual mobile operators are excluded. From these groups, also no socio-demographic information would be available. In addition to that, customers with an opt-out for such analyses were excluded, although these are quite rare (< 1 percent). More information on the data source can be found at TU Wien et al. (2021) and Reisch et al. (2021).

According to governmental statistics in Austria (Statistik Austria 2021), 87.8 percent of individuals between 16 and 74 access the internet via a smartphone in 2021. The share of users decreases with higher age from 98.9 percent (16–24) to 55.5 percent (65–74). However, the middle aged have still quite high rates of smartphone usage (45–54: 93.4 percent; 55–64: 79.5 percent). Thus, we only expect larger coverage issues in the group of those aged 60 and older. Smartphone usage is a bit higher for males (88.9 percent) than for females (86.8 percent), and this difference increases with age (males aged 65–74: 64.5 percent; females aged 65–74: 47,6 percent).

The localization method is based on the topology of the network, which corresponds to the location of specific broadcasting base stations. The MSP provides the localization information for each event based on the centroid of the network coverage simulation. Thus, localization is not based on the place of the base station itself but rather on the centroid of the area where the specific base station provides the best network coverage and thus mobile devices are most likely connected to it. The literature on device-based data mainly use six types of mobility metrics (e.g., Pappalardo et al. 2015; Gauvin et al. 2020; Pepe et al. 2020; Wu et al. 2021): number of transfers between areas or provinces, number of unique destinations, stay times at locations, travel times between locations, graphical mapping of movements (e.g., heat maps), and the radius of gyration (ROG). In our case, individual mobility is calculated using the radius of gyration, which is defined as the time-weighted root mean square distance between the center of gravity (i.e., the coordinate-wise time-weighted average) and each localization. Refer to the study by Heiler et al. (2020) for more information and see, for example, the work by Gauvin et al. (2020) for a similar approach to estimating human mobility using mobile phone data.

### Google’s GPS-Based Mobility Reports

Google’s original dataset is based on anonymized aggregated data from their “Location History” feature also used to show “Popular Times” for places in Google Maps, storing combined location data from the connected devices’ antennas and sensors (in addition to GPS, this technology uses data from WiFi, the mobile network connection, and other sensors). This feature is turned off by default and has to be activated by the users in order to be operational. In addition, the stored locations can be deleted in the user account’s settings. Hence, the included userbase may not match the socio-demographic characteristics of the overall population (Google 2021a, 2022a, 2022b). An inquiry to Google on further information to their data stayed unanswered. In particular, we asked for details on the calculation of their measure, categorization of mobility, discrimination between types of visit to an area (e.g., work versus shopping), discrimination between residents and visitors, as well as the share and socio-demographic distribution of users who opted in to their “Location History” feature. We hence cannot provide any more details on potential coverage error in the Google data other than the specifics of smartphone use, as already explained for the GSM data.

Google’s Community Mobility Reports feature six place categories: grocery and pharmacy, parks, public transport transit stations, retail and recreation, residential, and workplaces. The “grocery and pharmacy” category includes “places like grocery markets, food warehouses, farmers markets, specialty food shops, drug stores, and pharmacies” (Google 2022b). In the “parks” category, Google condenses “places like local parks, national parks, public beaches, marinas, dog parks, plazas, and public gardens” (Google 2022b). The “retail and recreation” category combines “places like restaurants, cafes, shopping centers, theme parks, museums, libraries, and movie theaters” (Google 2022b).

According to Google, the mobility data shows “how visits to (or time spent in) categorized places change compared to our baseline days” (Google 2021b). These 7 baseline days (for each day of the week) are the median values from the period Jan. 3 to Feb. 6, 2020. We use this information and recalculate the mobility estimates to represent percentage-point changes from our new baseline week (March 23–29, 2020). Despite the fact that Google provides no information on the daily absolute mobility of the baseline week, it is possible to fully account for changes of the baseline week. This is described in equation (1) calculating the daily average of mobility measure *y_k,i_*, where *x_k,i_* is the daily average of mobility measure for each day *i* and mobility place category *k* and *α_m,k_* is the mobility value of the new baseline day for each place category corresponding to the specific day of the week *m*. Afterward, we calculate the average of the weekly average mobility changes across the place categories and match this with the mobility estimates of the GSM and survey-based data.

(1) $$y_{k,i}=\frac{x_{k,i}-\alpha_{m,k}}{1+\alpha_{m,k}}$$

## Appendix B. Question Wordings

1. Frequency Respondents Leave Their Home by Reason of Mobility:

Have you left your home last week for the following reasons?

- Work
- Physical activity
- Meeting friends or family
- To buy medicine, receive medical care or to care for/help others
- To buy food
- To walk the pet
- Due to boredom or to enjoy my freedom
- Other procurements [always in penultimate position]
- Other [always in last position] *Matrix labels:*

- never
- on some days
- several times a week
- almost every day
- daily
- don’t know
- no answer

2. Frequency Respondents Stay at Home:

In the following, think about your personal behavior during the last week. Please indicate how often you have behaved as follows:

- You stay at home, except for necessities.

- almost always
- mostly
- sometimes
- rarely
- almost never
- don’t know
- no answer
