# Supplementary material for: Varieties of Mobility Measures: Comparing Survey and Mobile Phone Data during the COVID-19 Pandemic
Source: Public Opin Q. 2022 Dec 2;86(4):913–31. doi: 10.1093/poq/nfac042 (PMC9940778; doi:10.1093/poq/nfac042)
Supplement: nfac042_Supplementary_Data [file nfac042_supplementary_data.pdf]

Varieties of mobility measures: Comparing survey and mobile phone data during the COVID-19 pandemic

Supplementary materials

Fabian Kalleitner[*,1], David W. Schiestl[2], and Georg Heiler[3]

[*] *corresponding author*

[1] *University of Vienna, Department of Economic Sociology, Kolingasse 14-16, Vienna, 1090, Austria. (fabian.kalleitner@univie.ac.at)*

[2] *University of Vienna, Department of Economic Sociology, Kolingasse 14-16, Vienna, 1090, Austria. (david.wolfgang.schiestl@univie.ac.at)*

[3] *Complexity Science Hub Vienna, Josefstädter Straße 39, Vienna, 1080, Austria & TU Wien, Institute of Information Systems Engineering, Favoritenstraße 9-11/194, Vienna, 1040, Austria. (heiler@csh.ac.at)*

A Coverage, Panel attrition and Nonresponse 1

B Baseline week 6

C Mobility trends by subgroups 7

D Model based comparisons using linear regressions with time fixed-effects 12

E Results using a single item measure 15

F Changes between waves: Estimating the effects of the "Lockdown in the East" 16

A Coverage, Panel attrition and Nonresponse

To test whether panel attrition could have affected the survey mobility estimates, we check which respondents left the survey and whether our resampled subjects have similar mobility characteristics. To test this, we assume that those who left the panel and those resampled should have similar differences in mobility behavior compared to the panelists remaining in the survey. This is described in equation (1) calculating the mobility measure $mobility_i$ for each individual i. Where $part_{i,t-1}$ is a dummy variable indicating if a participant participated in the previous wave $t-1$ and $part_{i,t}$ is a dummy variable indicating if a participant was present in the current survey wave t. We calculate separate models for each possible pair of survey waves and compare the differences in the two dummy variables using a linear hypothesis test (Fox and Weisberg 2019). The basic idea is that those who dropped out and those who are resampled should have roughly similar differences in mobility compared to those who are present in both waves. Hence the two dummy variables should be roughly equal in size (formally $\beta_1 = \beta_2$).

$$mobility_i = \beta_1 * part_{i,t-1} + \beta_2 * part_{i,t} + \beta_3 * wave_{i,t} + \alpha + \epsilon_i \quad (1)$$

We find that the average difference between these two coefficients is quite small (0.0118) and get 16 statistically significant differences out of 215 p<0.05 and 29 for p<0.1. These shares are roughly equal to what we would expect because of false positives. This suggests that those joining or rejoining the sample have similar differences to those those who stay in the sample. Aggregate comparisons between waves should therefore provide quite consistent results if the assumption holds that the initial selection of participants into the survey is independent of patterns of change in the population. We note that these differences are not only small because similar panellists were re-sampled but also because the average mobility of those who dropped out did not differ much from those who

remained in the sample ($\overline{\beta_1} = 0.023$).

To check for potential biases because of item-nonresponse we first asses the shares of missing values for our aggregate mobility estimate and each mobility component. Afterwards we calculate binary logistic regression models with wave fixed-effects predicting the log of the odds of respondents providing missing values for our aggregate mobility estimate and each mobility component focusing on our main socio-demographic variables of interest in the manuscript (gender and age). On average, 15.90% of respondents each wave did not provide a substantive answer to every mobility component of our aggregate mobility estimate. The missing values are distributed quite equally across the components of the additive index with exception of the answer category 'other reasons' (work=3.20%, sport=2.22%, friends=2.35%, medicine=2.51%, food=1.78%, pets = 4.38%, pets=2.99%, boredom=4.43%, other=11.2%). Dropping the variable 'other reason' from our mobility index would reduce average misses for our aggregate measure to 9.58%. However, using this measure we receive nearly equivalent relationships over time between the survey mobility estimates and the estimates using GSM (Pearson's r=0.931) and Google GPS-based data (Pearson's r=0.928) compared to the results reported in the main manuscript using the full range of variables. This is because, on an individual level, the 'other' category does not add much variance compared to the index excluding this variable (Pearson's r=0.983) and it seems that those who did avoid responding to this category did not change differently from those who answered.

Focusing on regression results provided in Table S1, we find less missing values for middle aged respondents (45-59) compared to the youngest age cohort (14-29). Looking more closely at the patterns, we find some indications that people might use 'don't know' or 'no answer' options to indicate no mobility in this category, as, for example, males and those in the working age population have less missing values in the work category than others. Hence, we would suggest that structurally similar questions in the future could

profit from more clear indication of the answer option corresponding to zero mobility. We also find more item non-response between waves 13 and 19 and in wave 22. This could indicate some fatigue effects or that increased mobility during summer months lead some respondents to choose to avoid an answer here. All in all, however, the effects are rather small in size. For instance, the odds for middle-aged respondents (45-59) to have missing values are 12% smaller (exp(-0.127)=0.881) than for the reference group. Hence, ceteris paribus, we would expect 16.9% missing values for middle-aged respondents compared to 19.2% for respondents in the youngest age group (14-29).

Survey field periods were not strictly aligned with calendar weeks and the duration varied depending on how fast the sample reached the target of 1500 respondents per wave. We therefore had to decide upon reference points determining the week used to calculate averages of the mobile phone (GSM) and Google mobility (GPS) data. As the median response of the survey only varies from Friday to Sunday, with most field phases starting on a Friday, we decided to use the date of the median response as our indicator for the baseline week. Hence, if the median response in the survey was in week 19, we utilize the average daily movement measures of calendar week 19 in the other measures. This is a compromise due to the varying reference period in the survey ('last 7 days'). As this reference point depends on the date the respondents answered the survey, one would have to match the right mobility data to the right respondents, which is not possible in our case. A running average approach, matching every survey respondent (irrespective of other characteristics) with a corresponding 7-day average according to the day they finished the survey and calculating mean values afterwards, produces nearly no differences with regard to the main results of interest (Pearson's correlation coefficients: ACPP-GSM = 0.935; ACPP-GOG= 0.935; GSM-GOG= 0.955). We suggest that other studies might consider calendar-based reference periods like "mobility in the week of data x to date y" in their survey questions if field periods increase in time.

Table S1: Regression estimates of a binary logistic regression on respondents likelihood of item-nonresponse

	Item nonresponse:									
	Index	work	sport	friends	medicine	food	pets	bordom	shopping	other
	(1)	(2)	(3)	(4)	(5)	(6)	(7)	(8)	(9)	(10)
Gender: male	−0.038	−0.129	0.109	0.091	0.153	0.153	0.071	0.150	0.0005	−0.095
(ref. female)	(0.030)	(0.063)	(0.076)	(0.074)	(0.071)	(0.085)	(0.054)	(0.066)	(0.058)	(0.035)
	$p = 0.209$	$p = 0.042$	$p = 0.150$	$p = 0.216$	$p = 0.033$	$p = 0.072$	$p = 0.193$	$p = 0.023$	$p = 0.994$	$p = 0.008$
Age: 30-44	−0.025	−0.214	−0.029	−0.093	0.020	−0.140	−0.009	0.143	0.178	0.259
(ref:14-29)	(0.044)	(0.088)	(0.091)	(0.088)	(0.085)	(0.097)	(0.073)	(0.083)	(0.073)	(0.052)
	$p = 0.560$	$p = 0.015$	$p = 0.748$	$p = 0.288$	$p = 0.817$	$p = 0.150$	$p = 0.907$	$p = 0.084$	$p = 0.015$	$p = 0.00000$
45-59	−0.127	−0.443	−0.625	−0.624	−0.678	−0.898	−0.186	−0.352	−0.504	0.181
	(0.044)	(0.091)	(0.103)	(0.098)	(0.098)	(0.117)	(0.075)	(0.090)	(0.083)	(0.052)
	$p = 0.004$	$p = 0.00001$	$p = 0.000$	$p = 0.000$	$p = 0.000$	$p = 0.000$	$p = 0.013$	$p = 0.0001$	$p = 0.000$	$p = 0.001$
60-74	0.031	−0.071	−1.701	−1.975	−1.854	−2.478	−0.505	−1.121	−0.969	0.293
	(0.045)	(0.088)	(0.164)	(0.175)	(0.164)	(0.240)	(0.086)	(0.123)	(0.103)	(0.054)
	$p = 0.493$	$p = 0.421$	$p = 0.000$	$p = 0.000$	$p = 0.000$	$p = 0.000$	$p = 0.000$	$p = 0.000$	$p = 0.000$	$p = 0.00000$
Wave: 2	−0.158	0.470	0.366	0.055	0.161	0.602	−0.343	0.077		−0.077
(ref. wave 1)	(0.108)	(0.312)	(0.351)	(0.315)	(0.324)	(0.397)	(0.216)	(0.275)		(0.121)
	$p = 0.143$	$p = 0.133$	$p = 0.297$	$p = 0.863$	$p = 0.620$	$p = 0.130$	$p = 0.112$	$p = 0.780$		$p = 0.525$
5	−0.078	0.846	0.646	0.464	0.537	0.903	0.134	0.262	0.093	−0.180
	(0.107)	(0.294)	(0.334)	(0.290)	(0.301)	(0.379)	(0.193)	(0.265)	(0.215)	(0.125)
	$p = 0.463$	$p = 0.005$	$p = 0.054$	$p = 0.110$	$p = 0.075$	$p = 0.018$	$p = 0.489$	$p = 0.324$	$p = 0.667$	$p = 0.149$
8	−0.047	0.826	0.897	0.735	0.730	1.205	0.106	0.516	0.436	−0.015
	(0.106)	(0.295)	(0.322)	(0.277)	(0.292)	(0.365)	(0.195)	(0.253)	(0.201)	(0.121)
	$p = 0.659$	$p = 0.006$	$p = 0.006$	$p = 0.009$	$p = 0.013$	$p = 0.001$	$p = 0.587$	$p = 0.042$	$p = 0.031$	$p = 0.902$
11	0.120	1.045	0.956	0.565	0.757	1.237	0.237	0.512	0.585	0.170
	(0.103)	(0.286)	(0.319)	(0.285)	(0.290)	(0.364)	(0.189)	(0.253)	(0.196)	(0.116)
	$p = 0.244$	$p = 0.0003$	$p = 0.003$	$p = 0.048$	$p = 0.010$	$p = 0.001$	$p = 0.211$	$p = 0.043$	$p = 0.003$	$p = 0.145$
14	0.337	0.946	0.922	0.367	0.778	0.917	0.253	0.681	0.384	0.307
	(0.099)	(0.289)	(0.319)	(0.294)	(0.288)	(0.377)	(0.187)	(0.244)	(0.201)	(0.113)
	$p = 0.001$	$p = 0.002$	$p = 0.004$	$p = 0.212$	$p = 0.007$	$p = 0.015$	$p = 0.177$	$p = 0.006$	$p = 0.057$	$p = 0.007$
17	0.220	1.176	0.721	0.394	0.630	1.031	0.231	0.514	0.538	0.185
	(0.100)	(0.280)	(0.328)	(0.292)	(0.294)	(0.371)	(0.187)	(0.251)	(0.195)	(0.115)
	$p = 0.029$	$p = 0.00003$	$p = 0.029$	$p = 0.177$	$p = 0.033$	$p = 0.006$	$p = 0.217$	$p = 0.041$	$p = 0.006$	$p = 0.107$
20	−0.010	0.799	1.007	0.698	0.907	0.880	0.176	0.595	0.298	−0.145
	(0.104)	(0.294)	(0.315)	(0.277)	(0.283)	(0.379)	(0.190)	(0.248)	(0.205)	(0.122)
	$p = 0.927$	$p = 0.007$	$p = 0.002$	$p = 0.012$	$p = 0.002$	$p = 0.021$	$p = 0.353$	$p = 0.017$	$p = 0.146$	$p = 0.237$
22	0.297	1.493	1.075	0.811	0.943	1.013	0.392	0.754	0.676	0.214
	(0.100)	(0.272)	(0.313)	(0.273)	(0.282)	(0.373)	(0.183)	(0.242)	(0.192)	(0.115)
	$p = 0.003$	$p = 0.00000$	$p = 0.001$	$p = 0.003$	$p = 0.001$	$p = 0.007$	$p = 0.032$	$p = 0.002$	$p = 0.0005$	$p = 0.063$
Constant	−1.724	−4.209	−4.309	−3.899	−4.076	−4.535	−3.175	−3.878	−3.258	−2.277
	(0.080)	(0.251)	(0.277)	(0.234)	(0.246)	(0.325)	(0.150)	(0.208)	(0.165)	(0.093)
	$p = 0.000$	$p = 0.000$	$p = 0.000$	$p = 0.000$	$p = 0.000$	$p = 0.000$	$p = 0.000$	$p = 0.000$	$p = 0.000$	$p = 0.000$
Observations	32,552	32,552	32,552	32,552	32,552	32,552	32,552	32,552	28,166	32,552

Note: Item nonresponse: 0 = responded, 1 = avoided an answer ("Don't know" or "No answer"). Because of the space considerations we report only every third wave fixed effect. The model contains all wave dummies. Full results are available on request. Standard errors within parentheses.

While the underlying mobility estimates from the GSM and Google data have no upper limit, the estimate obtained from the survey is limited by the ‘daily’ answer option. This limit, however, should not lead to substantial measurement error due to data censoring since most people would not go to work, go shopping or do sports more often than daily. This notion is supported by the low shares of respondents choosing the answer category ‘daily’ (see Figure S1 below). Despite this limit in the survey, the self-reported data features more work-related mobility than Google’s Mobility Reports. This suggests that the issue of reduced variance in relative mobility due to an upper limit in the survey scale should be most suitable.

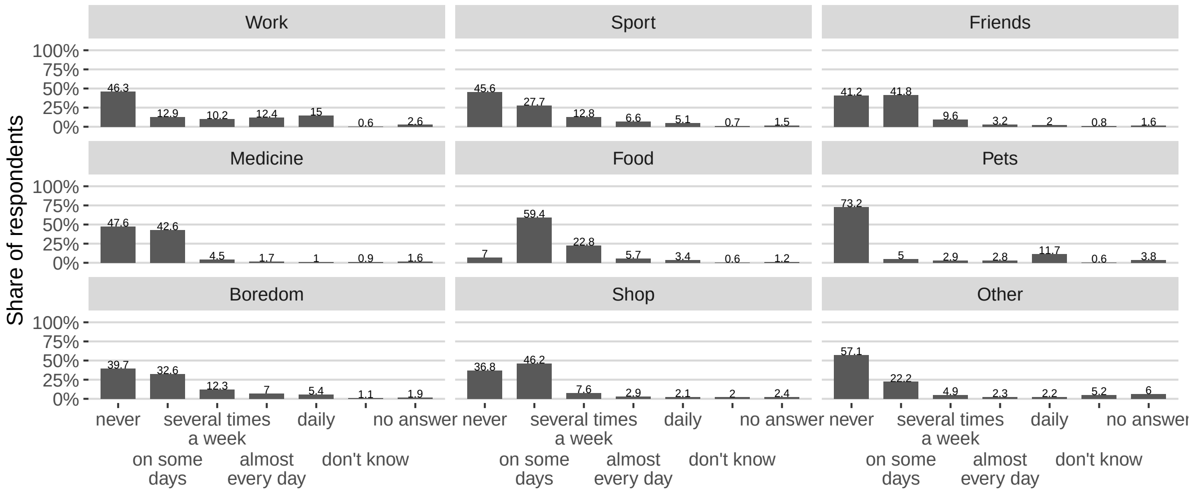

Figure S1: Answer shares of mobility items across all survey waves (by mobility category, N≈30.947 per item)

The survey question on whether respondents went out to ’buy non-food products’ was only asked from the fourth wave onward. This was because, until then, the governmental restrictions in Austria basically prohibited nonessential shopping. Hence, our additive index of mobility consists of 8 items until wave three, and 9 items afterwards. To account for this difference, we also exclude the place category ’retail & recreation’ for the Google estimate in the first three waves. Cronbach’s alpha for the survey index on mobility is

0.74 across all waves, indicating that the index represents a solid construct. Dropping the items on 'work' and 'walking pets' increases alpha by only 0.02, leading to the conclusion that the index from all items is reliable.

B Baseline week

Our baseline week (March 23–29) contains no major Austrian holidays or (non-pandemic related) school closures. Nevertheless, the choice of the baseline week can have substantial effects on our findings. To study how robust our results are to changes in the baseline week of our estimates, we calculated correlations and coefficients for different baselines. Correlation coefficients between the survey and the GSM estimates are unaffected by changes in the selected reference week. Differences only emerge with regard to Google's Mobility Reports, because this estimate relies on the assumption that every relative change in the mobility categories is equally important for relative changes in the overall mobility estimate. The other estimates calculate the aggregate indices first and then calculate the relative changes. We cannot use this approach for the Google data due to lack of data on absolute mobility. Changes in the reference week, however, only have minor consequences for correlations which vary between 0.864 [if wave 11 is used as the reference week] and 0.944 [wave 3] (for context, baseline wave 1 in main text = 0.936). The differences are larger with regard to the slopes of the time trends. The coefficients between Google's Mobility Reports and the survey mobility estimates vary between 0.749 [wave 13] and 1.771 [wave 4]. Coefficients also change when comparing survey mobility estimates and the GSM based estimate (2.542 [wave2] – 1.325 [wave 13]). These results can be explained due to the larger increases in the Google and GSM estimates compared to the survey measure. Thus, basing these estimates on these high values reduces variation over time in the Google and GSM datasets. This also explains

why subgroup differences remain largely unaffected. Irrespective of changes in the baseline week, we get larger coefficients for male than for female mobility and for middle-aged (30-44 & 45-59) compared to younger (15-29) and older (60-74) age cohorts. Coefficients for work are consistently smaller than those for the sum index and coefficients for shopping are consistently larger than those from the main index. The relation remains quite close to 1 for food and medicine (0.873 [wave 7] – 1.176 [wave 1]).

C Mobility trends by subgroups

Figure S2 shows the trends in relative mobility by subgroup throughout the pandemic analog to Figure 1 in the main manuscript. Overall gender differences are quite similar when comparing mobile phone and survey data. Both estimate slightly higher relative increases in mobility after the lockdown for women compared to men. While these differences remain quite stable in the survey data, they disappear after summer 2020 in the GSM data. Notably, mobile phone data suggests higher effects of the end of the lockdown in April and May 2020 and at the beginning of 2021. Absolute mobility trends are depicted in Figure S3. These trends show that, while mobility of women increased more than mobility of men, absolute mobility estimates are always higher for men than for women. Again, the differences in mobility estimates between the two data sources are quite small. The results are slightly different focusing on subgroup differences by age. Younger and older subjects generally show higher relative increases in mobility compared to others (see Figure S2). As suggested in the main text, this might stem from differences in the importance of work-related mobility in these age groups. Because work-related mobility remained important during the lockdown – as not all workers could work from home – the working age population had fewer opportunities to decrease their mobility. This higher mobility level decreases the potential for relative increases after the end of the lockdown,

as larger absolute changes in mobility nevertheless appear smaller in the relative mobility estimate. In line with this interpretation, the lockdown in the end of 2020 seems to have decreased these age differences. Again, increases in mobility at the beginning of 2020 and 2021 are more pronounced in the mobile phone data compared to the survey estimates. Figure S3 containing trends of absolute mobility estimates by age-group supports this explanation, as those in working age show lower increases in mobility in the first half of 2020 compared to the younger and older age groups. The absolute mobility estimates also indicate that mobility decreases with higher age both in the GSM and in the survey data.

Figure (S4) compares survey and Google based relative mobility trends. As noted in the main text, work related mobility is more pronounced in the survey estimate compared to the Google indicator. The difference points in the other direction for mobility related to 'shopping (other)' (=Google's 'retail and recreation' place category). Mobility in the category 'shopping (food and medicine)' is quite similar comparing Google's mobility reports and our survey based mobility estimates. In line with the idea that this mobility category captures essential parts of mobility, the graph indicates lower increases after the lockdown compared to the other mobility categories. Figure S5 focusing on differences in absolute mobility for the GSM and survey estimates shows that these relative differences emerge also due to large differences in the initial raw mobility values. As Google does not provide their absolute mobility estimates, the graph shows the relative changes based on Google's initial baseline. This indicates large relative decreases in the mobility related to 'shopping (other)'. While also low in the survey data ($\sim$0.1, indicating the average answer was between 'never' and 'on some days'), the survey mobility estimate increased less than the Google estimate. Besides higher initial values at the start of the pandemic in the survey estimate, this could also be because Google's 'retail and recreation' place category covers more mobility categories (such as visits to restaurants and movie theaters) than

our corresponding survey measure asking subjects to think of mobility frequency due to 'other procurements'.

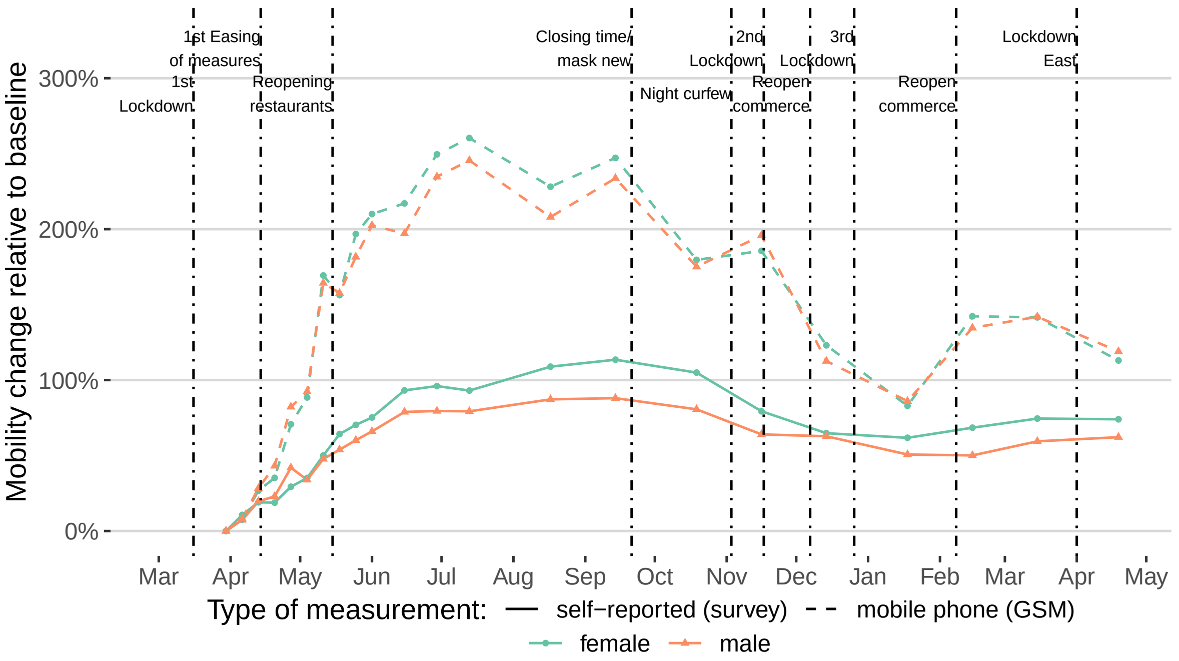

(a) By gender

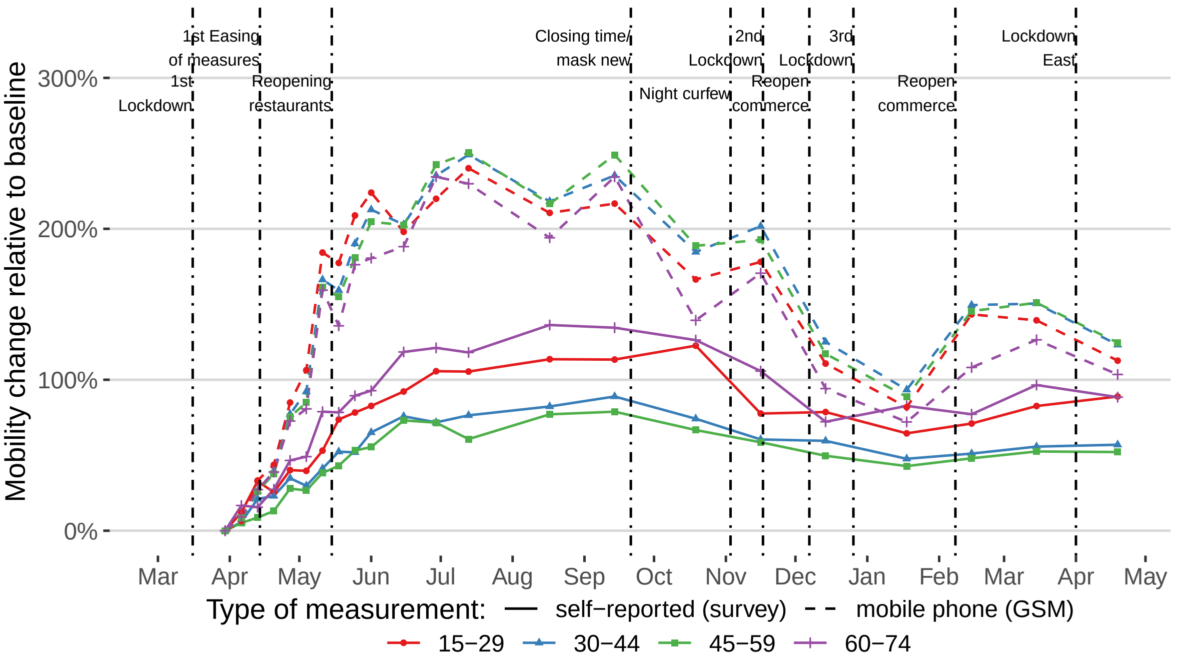

(b) By age group

Figure S2: Trends in relative mobility estimates (average by subgroup)

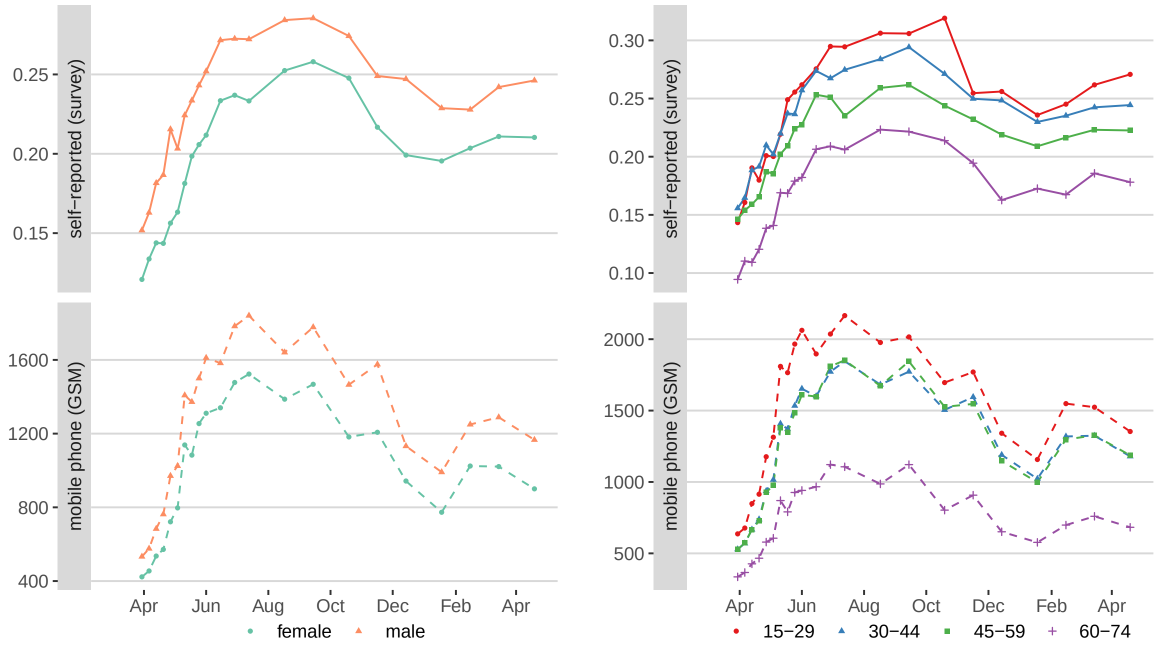

Figure S3: Trends in absolute mobility estimates (average by subgroup)

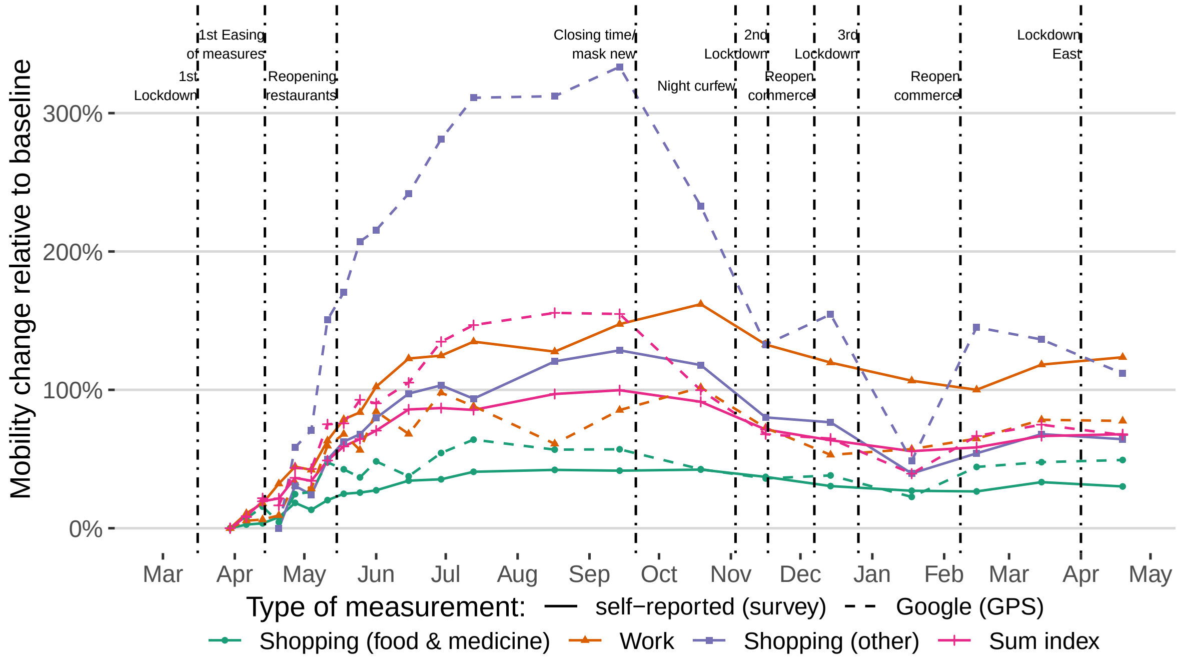

Figure S4: Trends in relative mobility estimates (average by subgroup)

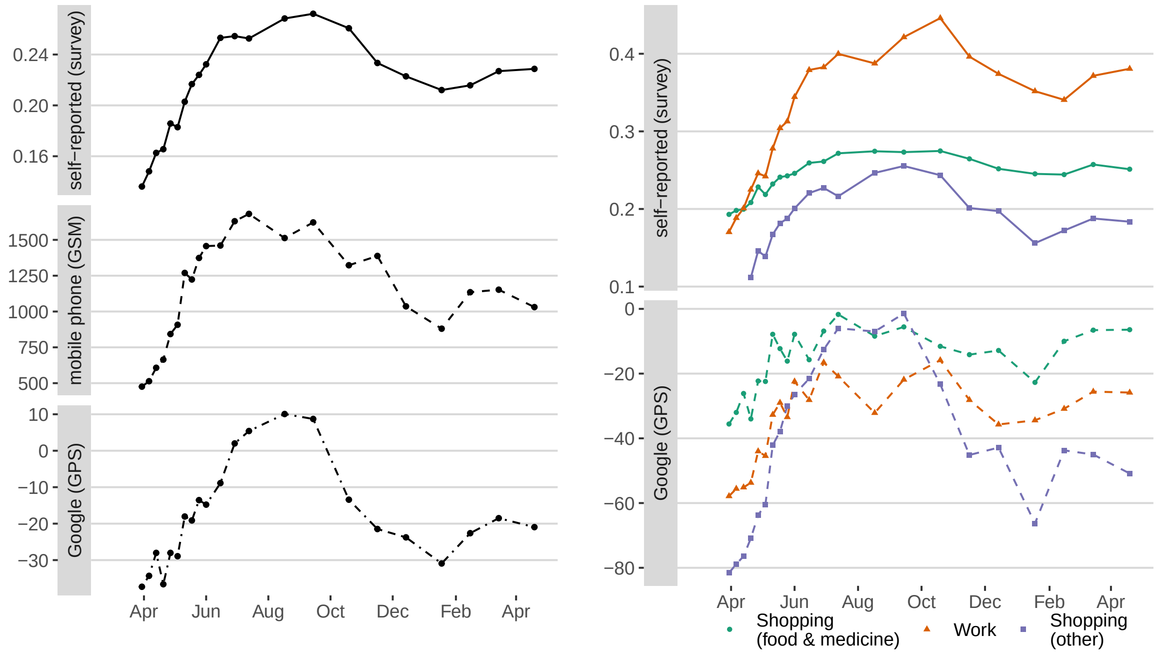

Figure S5: Trends in raw (Google) and absolute (survey) mobility estimates (average by mobility category)

D Model based comparisons using linear regressions with time fixed-effects

In addition to basic correlation coefficients, we also calculated regressions on aggregated mobility estimates with time fixed effects including dummy variables for the data sources (models 1-4), subgroups (models 3-4), and their interactions (to account for heterogeneous effects across data sources). The coefficient estimates of these models are presented in Table S2. Model 1 indicates larger variance in mobility over time in the GSM based mobility estimate compared to the survey. In model 2, we test whether this result differs depending on the stringency of governmental measures aimed to limit the spread of the virus. The estimated coefficients show that the differences are becoming smaller if stringency increases. This result suggests that the survey estimates do not suffer from considerable social desirability bias. Model 3 supports our results depicted in Figure

2 in the main manuscript. The coefficient estimates indicate only marginal differences between genders across the GSM and survey based mobility estimates. In addition, the relatively reduced mobility changes for middle age categories are less pronounced in the GSM estimate compared to those in the survey data. Similarly, model 4 supports the results displayed in Figure 3 of the main manuscript. Mobility estimates in the category 'shopping (food and medicine)' are smaller in comparison to the overall measure and larger for the mobility categories 'work' and 'shopping (other)'. The regression coefficients of the interactions indicate that variance over time in the category 'work' is smaller in the Google data compared to the survey data. This relationship is flipped in the case of mobility in the category 'shopping (other)'.

Table S2: Relative weekly mobility: OLS regression estimates

	weekly average mobility			
	(1)	(2)	(3)	(4)
Source: GSM (ref:survey)	0.819	2.709	0.658	
	(0.083)	(0.223)	(0.063)	
	$p < 0.001$	$p < 0.001$	$p < 0.001$	
Google (GPS)	0.160	1.018		0.160
	(0.083)	(0.223)		(0.099)
	$p = 0.063$	$p < 0.001$		$p = 0.111$
COVID-19 stringency				
GSM X stringency		−0.029		
		(0.003)		
		$p < 0.001$		
Google X stringency		−0.013		
		(0.003)		
		$p < 0.001$		
Gender: male (ref:female)			−0.235	
			(0.057)	
			$p < 0.001$	
GSM X male			−0.274	
			(0.057)	
			$p < 0.001$	
Age: 30-44 (ref:14-29)			0.081	
			(0.057)	
			$p = 0.157$	
45-59			−0.078	
			(0.040)	
			$p = 0.055$	
60-74			0.235	
			(0.080)	
			$p = 0.004$	
CSH X 30-44			0.274	
			(0.080)	
			$p = 0.001$	
CSH X 45-59			−0.081	
			(0.080)	
			$p = 0.317$	
CSH X 60-74			−0.005	
			(0.057)	
			$p = 0.935$	
Category: shopping (food/medicine)				−0.331
(Ref: Add. mobility index)				(0.099)
				$p = 0.002$
work				0.319
				(0.099)
				$p = 0.002$
shopping (other)				0.023
				(0.104)
				$p = 0.823$
Google X shopping (food/medicine)				−0.051
				(0.141)
				$p = 0.716$
Google X work				−0.496
				(0.141)
				$p = 0.001$
Google X shopping (other)				0.724
				(0.144)
				$p < 0.001$
Observations	66	66	352	173
Adjusted R^2	0.815	0.933	0.853	0.705

Note: Dependent variable = Relative weekly average mobility by source (model 1-2), by source, gender, and age-group (model 3), by source and mobility category (model 4). Standard errors within parentheses. Regressions include wave fixed effects not shown here.

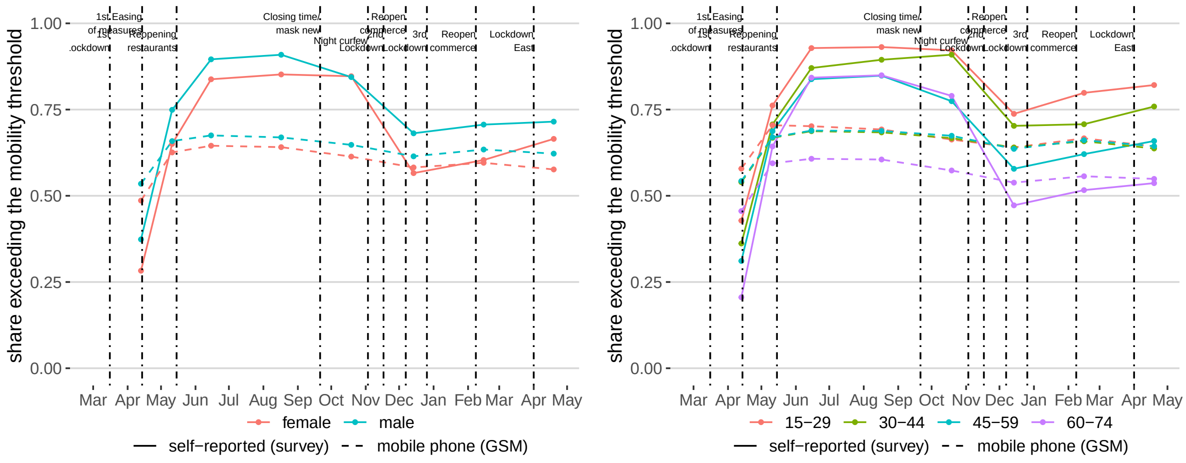

(a) By gender

(b) By age group

Figure S6: Trends in mobility estimates (average by subgroup)

E Results using a single item measure

To check the robustness of our results, we calculate similar analyses using a second mobility measure that utilizes respondent's self-reported frequency of leaving their home. This variable is only available every second wave. Specifically we compare the weekly absolute shares of people having a ROG of more than 500 meters to the absolute normalized survey wave average of a question asking for respondents' frequency of staying at home (we inverse this variable – question wording can be found in Appendix B). The results reported in Figure S6 and Figure S7 are quite similar to the ones using the additive index. Correlations are high and rather consistent. Also, the differences between genders are consistent across the estimates. Differences between age groups indicate that younger individuals are exceeding the mobility thresholds more frequently, which is particularly visible in the survey measure. The trends in the GSM measure might indicate that this indicator reaches upper bounds with lower age groups. However, also here, the elderly are clearly less mobile.

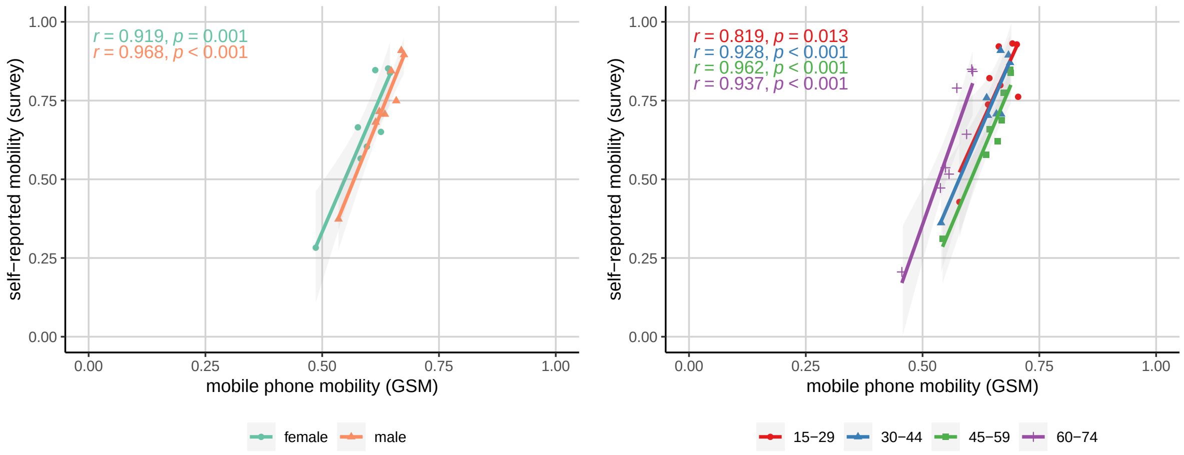

(a) By gender (b) By age group

Figure S7: Correlations of mobility estimates (average by subgroup)

F Changes between waves: Estimating the effects of the "Lockdown in the East"

One of the most prominent applications of mobility estimates during the pandemic is to calculate the effectiveness of different governmental measures targeted to reduce mobility and thus limit the spread of the virus. An important measure was the so-called "lockdown", which, in Austria, prominently named the closure of all shops and services except for "essential" retailers such as supermarkets and bakeries. While this measure was enacted nationwide most of the time, the infection situation in March 2021 led the regional governments of Burgenland, Lower Austria, and Vienna (all in the east of Austria) to pass a lockdown limited to those specific provinces starting on 1st of April. Burgenland returned to the nationwide rules on 19th of April, while Vienna and Lower Austria prolonged the lockdown until 2nd of May. We use this disparity within Austria for a Difference in Difference (DiD) estimation of the effectiveness of regional lockdowns on reducing mobility by comparing the mobility in Austria's east and west using all three different estimates. If estimates follow the same underlying trends in mobility, we would

expect somewhat similar sizes of the overall lockdown effect. Moreover, as possibilities for shopping were most affected by the regional lockdown, we would expect higher lockdown effects in this mobility category.

To estimate the DiD analyses we leverage information of respondents' home region in the survey data and utilize the fact that Google provides mobility estimates by region in addition to the estimates by country. Furthermore, the GSM data also contain information about the regional variation of the daily median ROG. In this dataset, the home location is calculated using the nighttime location of the mobile devices.

We assessed the validity of this DiD estimation strategy by first testing the critical parallel trends assumption. Figure S8 visually confirms that trends in average mobility estimates in all three datasets between March 2020 and March 2021 (i.e., pre-treatment) were rather similar in the treatment group (Burgenland, Lower Austria, and Vienna) and control group (Carinthia, Upper Austria, Salzburg, Styria, Tyrol, and Vorarlberg). Figure S9 shows that this also holds if we focus on mobility in the category shopping (other) in those data sources with distinguishable mobility categories.

Figure S8 shows an increase in mobility between March 2021 and April 2021 in the control group and a clear decrease in the treatment group within this time frame. Our DiD approach enabled us to estimate the causal impact of this lockdown treatment (Table 1). The constants describe the average mobility in the control group before the treatment. The coefficients suggest higher mobility changes from the baseline week in the control group ahead of the treatment in the GSM and the GPS (shopping) estimates compared to the survey estimate. We now focus on the interaction effect of treatment and time, representing our main estimates for the lockdown effect. Regression coefficients suggest that the main mobility measures utilizing Google's mobility reports (model 1) changed by 22.5 percentage points due to the lockdown. Utilizing mobility measures only for places related to retail and recreation (model 2), the coefficient increases to 72.4 percentage

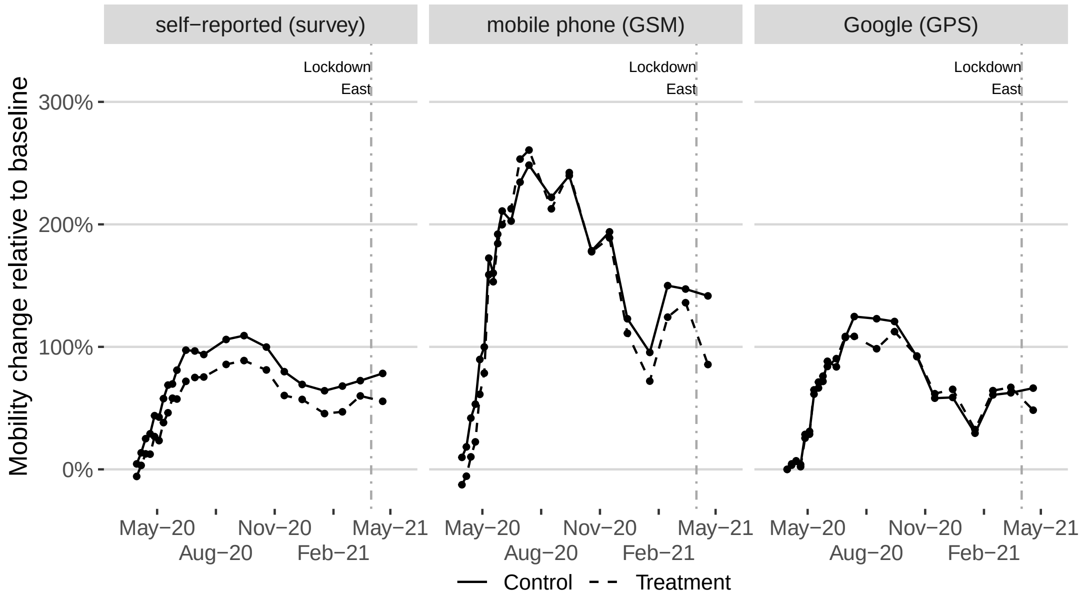

Figure S8: Mobility in treatment and control group over time (all)

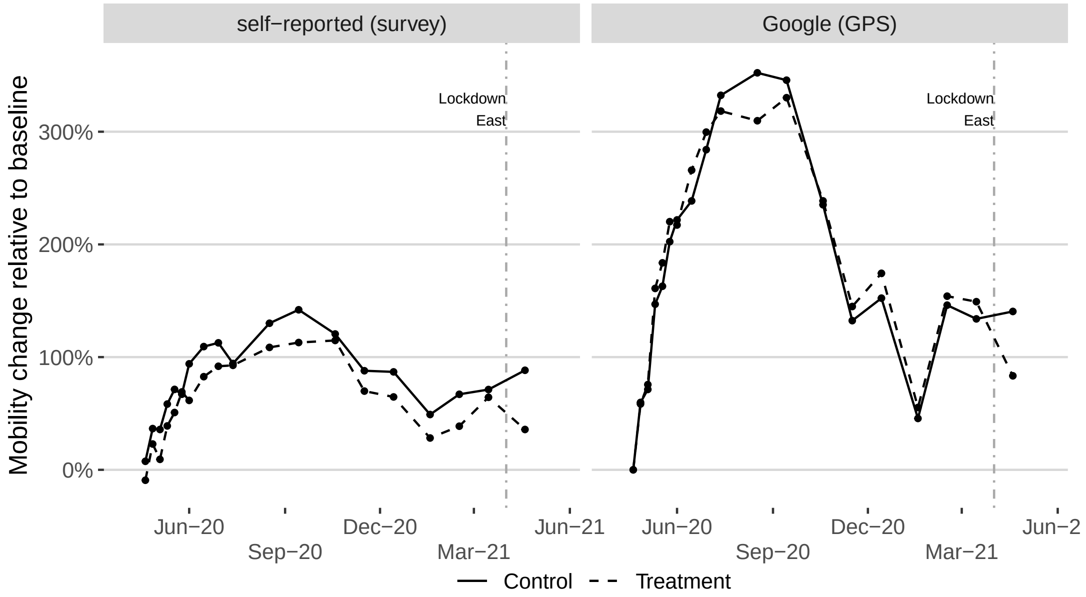

Figure S9: Mobility in treatment and control group over time (shopping -other)

points. This is also captured visually in the more pronounced variation between March and April of 2021 in Figure S9.

Structurally similar results are obtained when using survey data: model 4 suggests a reduction in mobility of 14.1 percentage points in the East due to the lockdown (not statistically significant at the .05 level). This effect strongly increases in model 5, focusing on the single mobility measure indicating how often people went shopping (47.8 percentage points, $p = 0.002$). The GSM data (model 3) show the most pronounced general effect of the Lockdown in Austria's east, indicating a reduction in mobility by 44.9 percentage points. Again, this shows the higher variation of this measure over time. Generally, however, all measures capture a reduction in mobility in Austria's East due to the Lockdown.

In addition, we note that differences between estimates could also stem from different definitions of an individual's home region. While cross-regional commuting traffic and tourism within Austria contribute to the mobility of an individual's usual home-region in the GSM and possibly also in the survey data, this is likely to be different for the mobility estimate reported by Google.

Table S3: Diff in Diff estimates of Lockdown East

	Mobility estimate:				
	GPS (all)	GPS (shopping)<br>Google	GSM<br>CSH	Self-reported (all)	Self-reported (shopping)<br>ACPP
	(1)	(2)	(3)	(4)	(5)
Treatment	0.045	0.153	−0.111	−0.074	0.060
				(0.062)	(0.109)
				$p = 0.234$	$p = 0.586$
Time	0.038	0.066	−0.056	0.035	0.164
				(0.059)	(0.105)
				$p = 0.553$	$p = 0.118$
Treatment X Time	−0.225	−0.724	−0.449	−0.141	−0.478
				(0.089)	(0.158)
				$p = 0.116$	$p = 0.003$
Constant	0.625	1.339	1.472	0.740	0.709
				(0.041)	(0.073)
				$p < 0.001$	$p < 0.001$
Observations	4	4	4	2,494	2,494

Note: As we only observe aggregate changes within the Google and GSM measures we cannot calculate estimates on the statistical variance of these effects with this data. Standard errors within parentheses.

Literature

Fox, John and Sanford Weisberg. 2019. "An R Companion to Applied Regression", Third Edition, London: Sage.
